# A microbial symphony: a literature review of the factors that orchestrate the colonization dynamics of the human colonic microbiome during infancy and implications for future health

**DOI:** 10.20517/mrr.2024.32

**Published:** 2024-09-24

**Authors:** Edward Horwell, Philip Bearn, Simon M. Cutting

**Affiliations:** ^1^Department of Biomedical Sciences, The Bourne Laboratory, Royal Holloway University of London, London TW20 0EX, UK.; ^2^Department of Colorectal Surgery, Ashford and Saint Peter’s NHS Foundation Trust, London KT16 0PZ, UK.

**Keywords:** Microbiome, paediatric, infant, inflammation, IBD, asthma, diabetes, newborn, microbiology, birth

## Abstract

Since the advent of new sequencing and bioinformatic technologies, our understanding of the human microbiome has expanded rapidly over recent years. Numerous studies have indicated causal links between alterations to the microbiome and a range of pathological conditions. Furthermore, a large body of epidemiological data is starting to suggest that exposure, or lack thereof, to specific microbial species during the first five years of life has key implications for long-term health outcomes. These include chronic inflammatory and metabolic conditions such as diabetes, asthma, inflammatory bowel disease (IBD), and obesity, with the effects lasting into adulthood. Human microbial colonisation during these first five years of life is a highly dynamic process, with multiple environmental exposures recently being characterised to have influence before the microbiome stabilises and resembles that of an adult at 3-5 years. This short period of time, known as the window of opportunity, appears to “prime” immunoregulation for later life. Understanding and appreciating this aspect of human physiology is therefore crucial for clinicians, scientists, and public health officials. This review outlines the most recent evidence for the pre- and post-natal environments that order the development of the microbiome, how these influences metabolic and immunoregulatory pathways, and their associated health outcomes. It also discusses the limitations of the current knowledge base, and describes the potential microbiome-mediated interventions and public health measures that may have therapeutic potential in the future.

## INTRODUCTION

The term “microbiome” was first coined by Professor John Whipps in 1988 to describe the microorganisms living in soil^[1]^. Colloquially, as well as in the scientific and medical lexicon, the term has anthropomorphically shifted, with the assumption of reference being to the human microbial ecosystem rather than the rhizosphere. The human microbiome is defined as the totality of microbial species found living in and on a human - that is, all the species of bacteria, archaea, fungi, algae, small protists, bacteriophages, and viruses, as well as extracellular DNA^[2,3]^. It also encompasses their “theatre of activity” - the secondary metabolites produced by these communities [e.g., toxins, lipopeptides, polysaccharides, signalling molecules, and other (in)organic matter] that often have important metabolic and immunogenic effects. The human microbiome is diverse, with populations differing greatly depending on anatomical location. As an example, the species found in the alar crease are significantly different from those in the inguinal crease, despite both being on the skin surface^[4]^.

The human-microbiome symbiosis should be viewed as one of co-evolution, with the microbiome aiding in the metabolism of food into short-chain fatty acids (SCFAs) (e.g., fermentation of the otherwise poorly digested plant polysaccharides and unhydrolyzed starches), the production of vitamins (e.g., thiamine, folate, riboflavin, pantothenic acid, biotin and vitamin K), and the control and competitive exclusion of pathogenic bacteria^[5]^. In response, the host provides a stable and rich environment with sophisticated immunological mechanisms to sense and control specific species^[6]^. Archaeological evidence from coprolites, dental plaque and tissue stored in permafrost have provided insight into how our microbiome has shifted away from that of the great apes to one of lower alpha diversity, with particularly decreased *Methanobrevibacter* and *Fibrobacter*, and significantly more *Bacteroides* species^[7]^*.* The recent (in evolutionary timescales) agricultural revolution, adoption of using heat to cook food, and Westernised diet (one of high meat and ultra-processed food consumption) have induced a rapid change in the makeup of our microbiome, which appears to have introduced mal-adaptions that are involved in diseases of modernity^[8,9]^.

The contemporary literature reports some ~3.8 × 10^13^ bacteria dwelling in the 70 kg “reference” male; however, it is the colon that is the true microbial powerhouse, making up 92% of the total microbiome^[10]^, and unless otherwise stated, hereon microbiome will be in reference to the human colonic microbiome. To regulate this, the gastrointestinal tract (GIT) maintains a fine balance of immune regulation and, accordingly, is the largest immune organ in the body^[11]^. This complex system involves the sensing of symbiotic species without stimulating the immune system, while detecting and mounting immune responses to specific pathogens^[12]^. If the interplay between this masterly inactivity and overactive defence mechanisms goes astray, there are numerous negative health-related consequences, ranging from inflammatory bowel disease (IBD), diabetes, cancer, obesity, cardiovascular disease, hypertension, depression, and anxiety (to name but a few)^[13]^. The “priming”, or education, of what the GIT immune system should consider a pathogen is, therefore, vital for future health^[12]^. The evidence suggests that this process is largely determined during the first five years of life; thus, it is of the utmost importance to fully understand and characterise the colonisation of a healthy microbiome during infancy.

Accordingly, this paper will review the latest literature on colonisation dynamics during the early years of life and what variables are at play. Specifically, we will seek out the mechanistic evidence for how bacterial species are transferred from one environment to the colonic microbiome of an individual, and discuss how this can be potentially utilised for public health. We will discuss any gaps and limitations of the scientific knowledge base and where the future of this new discipline is going.

## MICROBIOME VARIATION WITH AGE

The first months and years of life are a very dynamic period for the microbiome, with wide-reaching consequences for future health. Several large international longitudinal studies have identified three distinct periods of rapid and dynamic microbiomic progression^[14-17]^. These can be summarised as a developmental phase (the first 12 months of life) that is dominated by the effects of breastfeeding, a transitional phase (the second year of life, or the cessation of breast milk feeding) where the microbiome is highly plastic and adjusts to solid food, and culminating in a stable phase at approximately 36-60 months that closely resembles the microbiome of an adult^[14,18]^.

### Starting with a blank slate?

There has been a long-held belief that the human foetus develops in a sterile uterine environment^[19]^. The origin of this goes back to research that was unable to detect microbial life in the meconium, amniotic fluid, or placenta using traditional culture-based methods and microscopy^[20]^. Indeed, chorioamnionitis, an intra-amniotic infection typically secondary to E*.* coli or group B *streptococcus*, is associated with miscarriage, preterm birth, and increased neonatal mortality, ergo the presence of bacteria was deemed to be an abnormal phenomenon.

New techniques, however, such as next-generation sequencing (NGS), have transformed the detection and identification of microbiota. A growing body of evidence has raised questions over the “sterile uterus hypothesis”. Aagaard *et al.* were the first group to systematically describe a microbiome of placental samples from both healthy pregnancies and preterm births. They found a low-abundance but metabolically rich microbiome, which interestingly reflected the mother’s dental microbiome, raising the possibility of haematogenous spread from the oral cavity to the placenta^[21]^. Several other studies have since published evidence for distinct microbiomes relating to the placenta, amniotic fluid, and meconium in healthy neonates, resembling the faecal, oral, and vaginal microbiome of the mother^[22-24]^. Furthermore, animal models have shown a similar effect. A Spanish group inoculated pregnant BALB/c mice with an oral solution of a tagged mutant strain of *Enterococcus faecium*. This strain was then detected from samples of amniotic fluid taken at the time of caesarean section (CS), an effect not seen in the control group^[25]^. In our view, this is perhaps the most compelling evidence against the sterile uterus hypothesis.

This antithesis to the canon has caused controversy, however. Several papers have subsequently highlighted methodological errors pertaining to contamination from both the collection method (e.g., epidermal contamination from the puncture site for amniocentesis, or contamination during veterinary CS), and the typical 48 h delay in collecting meconium that might allow ex-utero colonisation to occur^[26-28]^. Accordingly, an international group repeated these experiments, with more rigorous controls for contamination, and failed to find evidence of intra-uterine colonization of the foetal GIT^[29]^.

An international consensus statement has made clear that the principal viewpoint of the academic community is still that of the sterile womb, citing, amongst several reasons, the high rates of false-positive results that exquisitely sensitive NGS can produce^[28]^. It should be noted that even in the event of true-positive results, bacterial DNA does not equate to living and metabolically active bacterial species that have colonised the foetal microbiome. Furthermore, there have been over seven decades of germ-free animal experimentation^[30]^, where mammals are born via CS at term. A valid assumption follows that if in-utero colonisation had occurred, this would have been detected in the numerous control groups during this time^[27]^.

Perhaps a nuanced slant to this, with increasing experimental data behind it, would be that immunogenic microbial products (bacterial DNA fragments, and metabolites) from the mother’s commensal microbiota are able to cross the placental barrier, giving the opportunity to educate the foetal immune system and promote immune tolerance before birth^[31-33]^. With the rapid progress in ex-utero embryogenesis technology^[34]^, it is conceivable that in the near future, an animal model will be able to categorically prove if these immunogenic microbial products have any demonstrable relevance to foetal immunogenic and microbiomic development. For now, the evidence is insufficient to move away from the orthodoxy of the sterile womb hypothesis.

### Birth

The very first moment of life has direct implications for the newborn’s microbiome. Delivery by CS or vaginal delivery (VD) has been shown to result in divergent neonatal microbiomes. This is believed to be due to the direct contact of the neonate with the maternal vaginal and perineal microbiome, allowing direct vertical transmission to occur. Although very few mechanistic studies have been performed, transmission to the neonatal colon is believed to be via the oral route, a process phrased as the “bacterial baptism of birth”^[35,36]^. An interesting physiological adaption arises during the third trimester that confers a survival benefit to the child. In response to increasing oestrogen and progesterone, the mother’s vaginal microbiome is altered, a decrease in alpha diversity occurs, and the abundance of *Lactobacillus* spp. increases^[37,38]^*.* These species produce lactic acid, decreasing vaginal pH and consequentially reducing acid-sensitive pathobionts such as Group B *Streptococcus*^[38]^, which carry a high rate of neonatal sepsis and mortality.

This “bacterial baptism” during VD is inherently absent in CS; instead, other microbial environments appear to play a role and lead to divergent microbiomes in the newborn. Multiple large studies have demonstrated that VD is associated with increased abundances of *Bacteroidetes* (particularly *Bacteroides fragilis*), *Bacillota* (chiefly *Lactobacillus* spp*.)*, and *Actinomycetota* (almost exclusively from the *Bifidobacterium* spp.), all of which are typically found in the vagina or recto-anal microbiomes. In contrast, CS has been associated with species typically found on maternal skin and microbiomes found in the hospital, with neonates having an increased abundance of *Bacillot*a (notably *Staphylococcus* spp*.*), *Firmicutes*, *Enterococcus*, and *Klebsiella*^[14,39-46]^.

An increasing number of epidemiological studies have demonstrated a correlation between CS and an increased risk for negative long-term infant health outcomes. These findings are diverse and not always consistent, but there are reported increases in risk for type 1 diabetes mellitus (T1DM)^[47-49]^, obesity^[48,50]^, asthma^[51,52]^, eczema^[53]^, respiratory tract infections^[54]^, coeliac disease^[55]^, IBD^[56,57]^, attention deficit/hyperactivity disorder^[58]^, and autism^[59]^. A recent meta-analysis of the paediatric consequences of CS, with over two million subjects, demonstrated increased risks for childhood asthma [OR 1.23 (1.14, 1.33)], T1DM [OR 1.07 (0.90, 1.27)], and obesity [OR 1.35 (1.29, 1.41) ]^[43]^. With more than 25% of births being CS in Europe and North America, the potential implications to long-term health are a significant public health concern^[60,61]^. However, it is still unclear what causes these phenomena. Several explanations have been proposed, from the lack of stress hormones during an elective CS to an absence of intraocular pressure being suggested to alter the inflammatory response of the newborn^[62]^. However, the leading hypothesis relates to the divergent microbiomes associated with these methods of delivery^[63]^. The first 24 h of life have been shown to be a prime opportunity for bacterial colonisation of the colon, in that the pH of the stomach is relatively neutral thanks to the ingestion of amniotic fluid in utero^[43]^. This has been shown to complement the vertical transmission during VD of *Lactobacillus, Bifidobacterium*, and *Bacteroides*. These have been shown to regulate the developing immune system, influence the concentration of natural killer (NK) cells, regulate the population of T-lymphocytes, increase the secretion of Immunoglobulin A (IgA) antibodies, and aid in the metabolism of human milk oligosaccharides (HMOs)^[64-70]^. Furthermore, *Bifidobacterium* has been shown in both human and animal studies to reduce the risk of necrotising enterocolitis in newborns^[71]^.

This microbiomic theory of disease has a large and convincing body of evidence to support it. However, when looking at the literature, there are two important deficits that require attention. Firstly, the majority of studies showing metagenomic differences in VD and CS children, while replicated many times, were not designed to determine why these differences occur. Are the differences related to the method of delivery itself - i.e., is the vaginal microbiome the only variable, or are there other unrecognised environmental factors at play? Studies traditionally have used genetic operational taxonomic units (OTUs) to show significant similarity in intra-mother-child dyads *vs.* inter-dyads^[72]^; thereby, evidence of VD vertical transmission is only strongly inferred. To the best of our knowledge, there have been no mechanistic studies (e.g., using tagged bacterial species) to definitively show colonisation from the vaginal microbiome to the neonate’s GIT. Indeed, *Bacteroides fragilis*, the species that correlates strongly with VD, is only found in 6.6% of the vaginal microbiome of pregnant women, suggesting inoculation from an environment other than the vagina^[73]^. Interestingly, studies using cultured vaginal bacteria from the mother, administered orally to the neonate shortly after CS, have reported both positive and negative effects on the infant’s microbiome compared to placebo, raising questions over the extent played by VD in forming the newborn’s microbiome^[74,75]^. In a similar vein, there is an increasing trend of the lay press in the advocation of “vaginal seeding”, inoculating a swab or gauze with vaginal fluid to transfer the flora to a newborn. More research is urgently needed to assess not only the efficacy of this but also to assess for any safety concerns, a view reflected by the American College of Obstetricians and Gynaecologists^[76]^. Another variable that may influence the paradigm is the routine use of preoperative antibiotics during CS, which has significant effects on the neonatal microbiome^[77]^. The evidence suggests that the routine use of pre-cord clamping antibiotics significantly reduces the abundance of bacteria, predominantly from reduced *Clostridium* spp*.*, an effect that can still be observed after one year of age^[77]^. Interest in antibiotic usage after cord clamping is increasing internationally, where the antibiotic compound does not reach the foetus. Randomised control trials of this method have shown that it successfully mitigates the effects on the neonatal microbiome^[77]^. Furthermore, a Swiss lead non-inferiority trial found that the technique had no negative effect on the mother (e.g., surgical site infection), and its routine adoption into clinical practice should be encouraged^[78]^. However, it should be noted that there is conflicting evidence on this; a Dutch group has recently reported no significant difference in microbiomic composition between infants born to CS with antibiotics given pre- *vs.* post-cord clamping^[79]^. Finally, maternal factors seem to influence microbiome dynamics, with one study showing a significant difference in children born by emergency CS compared to elective CS, suggesting that antenatal health regulates the microbiomic outcome^[80]^. Recent papers have shown that the differences in the microbiome of CS *vs.* VD are very short-lived. In one large longitudinal study using whole genome shotgun (WGS) metagenomic analysis, a more sensitive method compared to the commonly used 16S rRNA sequencing, the microbiome between CS and VD becomes indistinguishable at six weeks^[81]^. This provides a very limited window of opportunity for such a dynamic microbiome to determine long-term health outcomes for the child.

The second aspect to mention is the absence of prospective trials showing a pathogenic mechanism linking CS and negative long-term health outcomes. The evidence that does exist, exclusively from epidemiological studies, has been inconsistent in controlling for variables that are known to impact the health of a newborn. These include: (i) maternal and neonatal co-morbidity during pregnancy and after birth; (ii) rates of breastfeeding (known to be lower after CS); (iii) the impact of a hospital microbiome compared to a maternity unit / home birth; (iv) increased maternal age, all of which are known to be independently associated with paediatric health^[82]^. This list is not exhaustive and, importantly, neglects the unknown unknowns. Moreover, several of these variables, such as maternal co-morbidity, also independently increase the likelihood of having a CS, potentially compounding the effect.

### Breastfeeding (the developmental phase)

During this period, even when accounting for the mode of delivery, the most significant factor associated with the structure of the microbiome is the receipt of breast milk, either exclusively or partially^[14]^. Contrary to previously held dogma, human breast milk is not sterile^[83]^ and has been found to be rich in the genera *Bacillus*, *Bifidobacterium*, *Enterococcus*, *Lactobacillus*, *Lactococcus*, *Weisella*, *Staphylococcus*, and *Streptococcus*^[84-87]^. One paper has recorded a median bacterial load of 10^6^ cells/mL of breast milk, suggesting that exclusively breastfed (EBF) infants consume approximately 8 billion bacteria per day^[88]^. The origin of the lactational mammary duct microbiome is complex and likely multifaceted. DNA sequencing of the milk has demonstrated species otherwise typically found in the oral cavity and maternal skin. It is reasonable to assume colonisation occurs during suckling of the infant as well as from direct contact with adjacent skin microbiomes. This, however, does not explain the presence of obligate anaerobes found in milk that are prototypical of an adult’s colonic microbiome - *Enterococcus*, *Bifidobacterium*, *Bacteroides*, *Clostridium*, and *Faecalibacterium*^[89,90]^*.* Recent evidence has suggested an entero-mammary pathway whereby bacterial cells from the colonic lumen are phagocytosed by dendritic and CD18^+^ cells, taken into the systemic lymphatic and cardiovascular circulation and then deposited in the mammary gland^[91]^. This mechanism is analogous to the established entero-mammary circulation, where B cells migrate to the mammary epithelium and release secretory (S) IgA into the milk supply^[92]^. If this mode of bacterial translocation is confirmed with mechanistic studies, it would provide a clear route for the initial colonisation of strict anaerobic species that are not typically found on surface environments, and the absence of this mode of transmission could have potentially profound consequences on the development of the colonic immune system. This may go some way to explain how certain bacterial species that are exquisitely vulnerable to atmospheric conditions can reach the human GIT. For example, *Akkermansia mucinophilia*, a species that is inversely related to IBD and obesity, is notoriously difficult to culture and survives for very short periods under atmospheric conditions^[93]^; thus, it has no known extra-colonic environmental niche to provide a stable supply for colonisation. A recent study has detected this species in the colostrum and milk supply from mothers^[94]^, perhaps being a key moment for this, and similar species, to establish themselves for permanent colonisation of the newborn’s GIT. It has previously been demonstrated that maternal diet affects the milk microbiome^[95]^, and this may be a branch of interesting future research to develop specialised maternal diets to modulate the developing neonatal microbiome with the aim of improving long-term health outcomes.

Infants fed exclusively formula milk (FM) do not receive these bacterial species. Accordingly, they have remarkably different microbiomes to EBF infants. One meta-analysis of 684 infants^[96]^ from five countries showed an increased abundance of *Bacteroides*, *Eubacterium*, *Firmicutes,* and *Veillonella* in formula-fed babies. EBF infants had a lower alpha diversity due to the increased abundance of the genus *Bifidobacterium*. This increased *Bifidobacterium* abundance is associated with a growth in microbial metabolic pathways favouring lipid and vitamin metabolism. Several studies have taken these findings, along with the increased *Bacteroides* [associated with increased body mass index (BMI)], to suggest that FM may cause obesity, diabetes, and other adverse health outcomes in adulthood as a result of the altered nutrient metabolism in the colon^[97,98]^. Furthermore, the differences in microbiomes between infants who are EBF and FM appear to be subtly more pronounced if the mode of delivery was CS. The evidence suggests that the relative absence of anaerobes (typically found in the microbiota of CS-delivered children) is exacerbated for infants who are then subsequently on FM^[99]^, particularly in *Proteobacteria*, suggesting an increased importance of EBF after CS. Another interesting finding is the microbiomic difference between infants who are EBF and those who receive human breast milk from a bottle^[100]^. For instance, while metagenomic studies of fresh milk and milk stored at 4 °C show no significant difference, there is a significant reduction in viable bacterial species - for instance, *Lactobacillus* and *Streptococcus* spp., possibly explaining the differences in the microbiome of these two cohorts of children (i.e., fresh human breast milk *vs.* refrigerated human breast milk)^[101]^.

Human breast milk has a complex mixture of essential macronutrients that help a newborn’s normal development, as well as acting as a prebiotic. It also contains immunogenic compounds, such as SIgA (and, to a lesser extent, IgM and IgG), which provide a degree of passive immunity for the newborn to specific pathogens^[102,103]^. As with the mode of delivery, the difference between the microbiomes of children who are formula- *vs.* breast milk-fed has been proposed as a causative factor for several associated pathologies. Voluminous epidemiological studies and the cumulative power of their meta-analyses have shown clear associations between breastfeeding and beneficial health outcomes. The comprehensive list includes a reduced risk of: asthma^[104]^^*^, eczema^[104]^, respiratory infections^[105]^^*^, IBD^[106]^^*^, infectious diarrhoeal diseases^[105,107]^^*^, leukaemia^[108]^^*^, diabetes melitus^[109,110]^, obesity^[111]^^*^, acute otitis media^[112]^, dental caries^[113]^, and general intelligence^[114]^. Several of these papers have gone further to demonstrate a protective dose response to the aforementioned (^*^) pathologies, giving credence to a mechanistic link. The precise time when the benefits from EBF start to taper off is not clear, with conflicting evidence between four and six months as the cut-off times. A Cochrane review found that the balance of evidence supports EBF for six months, which is reflected in the international guidance^[115-117]^.

### After breastfeeding (the transitional and stable phases)

At approximately 12 months, coinciding when the majority of infants have completed weaning, the microbiome changes sharply. The cessation of EBF and the introduction of solid food results in a shift towards species that are predominantly fermenters of plant-based polysaccharides and fibres, converting them into SCFAs, such as butyrate^[17]^. SCFAs are known to be essential for colonocyte metabolic health, as well as having anti-inflammatory and anti-carcinogenic properties^[118]^. This is characterised by an increase in *Bacteroides*, *Clostridium*, *Enterobacteria*, *Enterococci*, and *Streptococcus*^[17,119]^. Infants with a high-fibre diet display a considerable increase in *Bacteroidetes*^[120]^. In the distal colon, these phyla further contribute to the host through the biosynthesis of vitamins and essential amino acids^[119]^. It has been suggested that a westernised diet during this period reduces the populations of *Bacteroidetes* and *Prevotella*, which may lead to dysbiosis and partly explain the increased risk of IBD^[119]^. Interestingly, this shift in the microbiome is most apparent when breastfeeding stops, rather than when food is introduced^[17]^. This effect is less evident in formula-fed infants as their diet already included complex nutritional compounds that had resulted in a more diverse microbiome earlier in life^[121]^. After 12-24 months, this highly plastic phase starts to stabilise and resembles the microbiome of adults. It is during this period that other environmental stimuli start to have an impact on the microbiome.

## ENVIRONMENTAL FACTORS

### The home environment

Studies have shown that modern humans spend just under 90% of their time indoors^[122]^. It, therefore, logically follows that the home environment would have a significant environmental influence on the microbiome of children. The microbiome of modern homes, compared to pre-industrial home environments that were intertwined with soil, plant and animal microbial life, is low in both diversity and abundance. This is exacerbated by the scarcity of nutrients, modern surface materials, and chemicals used to keep homes clean. These selective pressures result in a relative increase in environmental exposure to *Basidiomycota*, *Deinococcus*, *Enhydrobacter*, *Micrococcus*, *Paracoccus*, *Staphylococcus*, and *Streptococcus*^[123,124]^. It has been suggested that indoor plants go some way in increasing microbial diversity and provide a protected environment for microbial life, allowing stabilisation of the home microbiome, although there have been no epidemiological studies assessing if this makes any difference to human health^[125]^.

Therefore, it is perhaps surprising that despite the significant time spent in the home environment, several studies have found that it is only responsible for a small proportion of the variation seen in the infant’s microbiome^[126,127]^. One study looked at adopted children and compared their microbiome to their genetically unrelated siblings and found that the shared home environment explained 6% of the variance^[128]^*.* The number of occupants in the shared house (i.e., how many siblings or parents cohabit in the house) has also failed to provide consistent results for any effect on the microbiome, although the presence of older siblings appears to alter the rate of maturation of the infant microbiome^[127,129]^. To add to the complexity that researchers face in trying to elucidate environment variables that affect microbiomic dynamics, twin studies have demonstrated increased concordance among monozygotic compared to dizygotic twins, showing that there is a genetic influence on the microbiome^[130]^. For example, the LCT, Fut2, irak4, and Nod2 genes have been directly linked to an alteration in the abundance of *Bifidobacterium*, *Lachnospiraceae*, *Roseburia*, *Bacteroidetes*, and *Firmicutes*, respectively^[131-134]^. This, in part, explains why genetic mutations (e.g., in Nod*2*) causing perturbations in the microbiome might be pathogenic for IBD^[132,135]^.

Although no consistent effect has been found on the microbiome of adults^[136]^, household pets modulate the microbiome of children^[137]^. Specifically, *Bifidobacterium* is increased in children with household dogs^[137]^. Having a dog in the house during infancy reduces the risk of childhood asthma, an atopic disease that appears to have an inverse relationship with *Bifidobacterium*^[137]^. A recent meta-analysis using cohort studies of children with household dogs adds weight to this, with a risk reduction for asthma of 0.85 (95%CI = 0.73-0.97)^[138]^. Interestingly, there appears to be a “dose response” with this association, with one study showing that two or more dogs had a greater effect than a single dog^[139]^. While we believe that the prevailing evidence supports household dogs being protective, albeit with potentially a small clinical effect, there is still conflicting evidence in the literature. As an example, one meta-analysis using a mix of cohort and case-control data came to an antithetical conclusion^[140]^.

### Day care

Children attending day care facilities are exposed to a group of peers with different microbiomes during their dynamic period of colonisation. One study found that compared to homeschooled children, day care resulted in a significantly more diverse and abundant microbiome, with an increased abundance in *Bacteroidetes*, *Firmicutes*, *Lachnospiraceae*, and *Ruminococcaceae*^[141]^. Perhaps unsurprisingly, the same group found that the microbiomes of peers at individual day care centres become more similar to each other compared to children in separate facilities. Previous studies have noted that the mixing of children in day care facilities is inversely related to childhood diabetes, suggesting that the microbiome might influence the pathogenesis^[142]^. Taken together, it appears that the mixing of children from outside of the household has a larger effect on the microbiome than mixing within the household; this is presumed to be due to the relative homogeneity between parents and offspring compared to the more diverse microbiomes of multiple classmates.

### Geographical proximity to the natural environment

There is significant heterogeneity between global populations^[143]^; for example, populations living in agrarian societies generally have a higher abundance of *Bacillus*, *Clostriales*, *Prevotella*, *Proteobacteria*, *Ruminobacter*, *Spirochaetes*, and *Succinivibrionaceae*, while that of modern urban populations have higher *Bacteroides*, *Bifidobacterium*, *Blautia*, and *Firmicutes*^[144-146]^. However, elucidating if this is due to the geography of nations (e.g., urban USA *vs.* rural Malawi) or their respective diet, cultural habits, and climate, among others, has proved hard to determine. One review has noted that even within individual nations, urban dwellers are more likely to be born via CS, less likely to have been EBF, and more likely to have been exposed to microbiome-altering medications (e.g., antibiotics) during childhood, making the distinction between their rural counterparts more complicated^[147]^.

It is, however, clear that when looking at the granular level, i.e., intranational, the geography of where children live has significant impacts on the microbiome and health outcomes of children. Numerous epidemiological studies have shown that children who grow up in rural locations have a significantly lower probability of IBD, asthma, and diabetes in later life^[148-150]^. The mechanism of this association has only recently started to become causally linked to the microbiome. As an example, one study looked at children growing up near (< 500 m) rural environments and compared their microbiome to those in urban areas, finding that the proximity to nature increases the abundance of *Proteobacteria* and *Verrucomicrobiales*, genera known to be beneficial symbionts and from the microbial rich soil^[151]^. These findings have been replicated with children growing up on farms^[14]^. A Finnish study found that children who grow up with this rural microbiome have reduced inflammatory response to bacterial cell wall antigens, with reduced expression of the pro-inflammatory cytokines IL-1β, IL-6, and IL-12^[152]^. Moreover, an elegantly designed study has demonstrated a linear “dose response” to a child’s proximity to greenspaces, showing that infants growing up in urban areas near a greenspace (e.g., a park) have a degree of protection to IBD, and as the distance to urban greenspaces increases, this protective effect decreases^[153]^.

The soil found in rural areas, particularly forest floors, appears to be a crucial reservoir for beneficial microbial species that modulate the immune system, particularly in terms of atopic disease and IBD^[154,155]^. Indeed, soil is the most microbially rich substrate to be described^[156]^ and the soil-dwelling species (e.g., *Bacillus*) have been identified as candidates for Rook’s old friends hypothesis^[157]^. This theory posits that we have co-evolved with environmental microbiomes typically encountered as forest hunter-gatherer omnivores, and these microbial species drive immunoregulatory and metabolic pathways^[158]^. Without these microbial populations, our GIT has an increased risk of dysregulation and results in the chronic inflammatory conditions often seen in the urbanised West. This does not explain the whole pathogenesis, but the mounting evidence does suggest it at least explains part of it^[159]^. Furthermore, animal studies have shown that exposure to soil during early life impacts the metabolome of the microbiome, increasing beneficial SCFAs, an effect that persists into adulthood^[160]^. Our group has recently shown that exposure to a strain of *Bacillus velezensis*, a prototypical soil dweller that produces bioactive cyclic-lipopeptides, can modulate the immune system in a beneficial way that ameliorates acute ulcerative colitis^[161]^. One group from Finland has gone one step further and designed an interventional trial for children aged 3-5 growing up in urban day care centres. These children were exposed to a specific rural microbiome by covering their outdoor areas with soil transplanted from a forest. They found this modulated the children’s microbiome with a particular increase in *Proteobacteria*, and noted an increase in plasma IL-10, a potent anti-inflammatory cytokine^[162]^. How these soil bacteria colonise the human GIT is poorly understood. It has been hypothesised that we are exposed to them by eating unwashed food, and by direct contact with the soil (e.g., playing in a field)^[163,164]^. The recently characterised aerobiome may also be responsible, at least in part, for colonisation dynamics in infants. As air blows over the ground and vegetation, microbial species are picked up and transported, and studies have shown significant differences in urban and rural aerobiomes, with rural aerobiomes having a greater abundance particularly in *Bacillus* and *Proteobacteria* than urban ones^[165,166]^. One group has experimentally demonstrated how exposure to a soil-based aerobiome might modulate the microbiome^[167]^. This might explain the linear proximity effect described earlier for children living in cities but near greenspaces, although more research on this new science is needed.

### Antibiotic exposure

We have already discussed how antibiotic use during the antenatal period can influence the neonatal microbiome. Perhaps unsurprisingly, antibiotic exposure in early life, either administered directly, or indirectly via breast milk if the mother is taking antibiotics, also reduces the alpha diversity of the microbiome^[168]^. Surprisingly, however, this effect can still be found long after the child stops taking antibiotics. Unlike in adults, where studies have shown that the microbiome recovers approximately two weeks after stopping antibiotics^[169]^, children have a much less stable microbiome during their developmental and transitional phases and can take months to years to recover^[170,171]^. Specific changes to the microbiome from antibiotic use in children are characterised by reduced *Actinobacteria*, *Bacteroidetes*, *Firmicutes*, and *Verrucomicrobia* populations, which are significant producers of beneficial SCFAs^[172]^. These perturbations in the microbiome in early life have been associated with childhood obesity, IBD, asthma, and allergies^[173-176]^. Furthermore, there are extensive mechanistic data from animal models supporting the causal link between the dysbiosis in the microbiome and subsequent pathology^[177,178]^. In contrast, certain bacterial cadres have been shown to increase in abundance after antibiotic use, such as E. coli, E. cloacae, K. pneumoniae, C. difficile, *Erysipelotrichaceae* spp*.*, and *Enterococcus* spp*.*, which are considered pathogenic and are associated with infective diarrhoeal disease, opportunistic infections, and IBD ^[177,179,180]^. The use of antibiotics also alters the resistome, in that it increases the abundance of antimicrobial-resistant species, raising the risk of future clinically significant antibiotic-resistant infections^[180]^. Certain multi-strain probiotics may be suitable for rapid replenishment of the microbiome after antibiotic use, with one randomised control trial showing a significant reduction in antibiotic-related diarrhoea in children under the age of 18^[181]^. Furthermore, evidence summarised in a recent Cochrane review suggests there may be a utility for probiotic use in necrotising enterocolitis when used in combination with standard care^[182]^. While no powerful study has looked at the long-term outcomes of children taking probiotics or live bacterial therapeutics (LBT), there is evidence that *Lactobacillus*-, *Bifidobacterium*- and *Bacillus-*based LBTs can beneficially modulate the microbiome and reduce atopy, respiratory, ear and gastrointestinal infections^[183-187]^. While antibiotic use has a large and perhaps obvious impact on the microbiome, it should also be noted that the use of gastric acid-suppressing medications (e.g., proton pump inhibitors and histamine receptor H2 agonists antagonists) that are used in children with symptomatic reflux also influences the colonisation dynamics of infants. They have been shown to increase the abundance of *Enterobacteriaceae*, *Clostridium*, and *Haemophilus*, while reducing *Firmicutes* and *Lactobacillus*^[188,189]^.

### Gaps in the literature and future research potential

This literature review has described the latest evidence on numerous variables that impact the orchestration of the infant microbiome during the highly dynamic period of the first half decade of life. It has documented the evidence for health implications of these variables, and sought clarity on research for mechanistic causation.

The body of evidence on this topic is large and growing rapidly. PubMed has over 10,500 results for “paediatric microbiome” from the last decade, and more than half of these were published in the last three years alone. A significant proportion of these academic papers are based on epidemiological data. Accordingly, for many of the results presented, causality can only be inferred. To address this, several research groups have used animal models to control for environmental stimuli. These animal models require meticulous methodology, and often employ germ-free or gnotobiotic species that are greatly divorced from natural conditions. As such, the applicability to general human microbiomic dynamics is hard to fully establish. Consequently, and despite significant research, there are two important deficits in our understanding of this vital period of microbial colonisation: (i) the causal relationship from a specific environmental stimulus to establishing a colony in the GIT; and (ii) how specific microbiomes influence long-term health.

Addressing the first point, definitively showing that exposure to environment A leads directly to microbiomic outcome B, might appear pedantic given the voluminous observational data. However, without knowing specifically how species rarely found in the mostly aerobic outdoor environment colonise the infant GIT, such as *Akkermansia Mucinophilia,* devising future interventions to aid the developing microbiome is going to be problematic and imprecise. This can be seen contemporarily in novel vaginal seeding techniques used after CS, which have so far failed to convincingly replicate the microbiome of children born via VD. The entero-mammary pathway of translocating bacteria from the maternal colon to breast milk appears to be a fruitful avenue for future research. Furthermore, while the widespread use of 16S genomic profiling has transformed our understanding and ability to measure the microbiome in a cost-effective way, it does not provide the entire picture. For example, 16S RNA is not sufficient for fungal or viral profiling, and both methods fail to give clarity on strain-specific population changes. As an example, it has been estimated that 75% of our microbiome, at the bacterial strain level, has yet to be characterised, colloquially known as dark matter^[190,191]^. Furthermore, metagenomics is not specific to the metabolome of the microbiome, which provides the mainstay of bioactivity. The fine details of this are beyond the scope of this paper, but a recent review covers the topic well^[192]^. New technologies are showing promise to give researchers the tools to track specific strains that might produce clear evidence of transfer from one environment to the GIT, and the variation in metabolic output from different strains (e.g., WISH-tagging); however, economic viability has limited widespread use, and they have not yet been employed in this specific field^[192,193]^. The use of genetically tagged bacteria has been used for several decades as a cost-effective way of tracking microbiome dynamics in adults across different disciplines^[194,195]^. To date, and to the best of our knowledge, there has only been one study using this technique on the infant microbiome^[25]^. More studies using these techniques are needed to clarify with precision modes of microbial transmission. Addressing the second point, observational studies that demonstrate differences in health outcomes all suffer from well-established biases and the inability to control for unknown confounders. As already highlighted, these studies can, therefore, only infer causality^[196]^. Due to the nature of the subject matter (the paediatric population), it is not surprising that randomised trials have not been performed. Ethical approval for a randomised trial into CS *vs.* VD would be near impossible to justify, let alone finding volunteers for recruitment. Animal models have been, therefore, crucial for deepening our understanding of how differences in the microbiome affect metabolic and immunogenic health. Despite the critiques of observational data listed above, pragmatism and the highly consistent and reproducible evidence of associated long-term health benefits for the infant should, and do, guide public health organisations as well as professional bodies to advocate for VD over CS, and for exclusive breastfeeding for at least six months. It should be noted that there are further benefits to breastfeeding that have not been discussed, such as the nutrition provided by breast milk in nations that have food insecurity or unsanitary water conditions^[197]^; the protective effects of breastfeeding on the mother’s risk of breast cancer, etc.^[198]^, but these are beyond the scope of this paper.

### The critical window of opportunity

It is clear that the infant microbiome is influenced by numerous variables in a highly orchestrated and dynamic ecosystem that follows a similar pattern across different global populations. CS is associated with an altered microbiome compared to VD, although the mechanism of this difference is not fully established. The largest single effector of the microbiome is breastfeeding, with very clear evidence that once this ceases, the microbiome of the infant quickly starts to change to that of an adult. During this time, environmental stimuli also become more apparent (e.g., diet, living in a rural environment, going to day care, etc.) and the microbiomic differences from variables seen earlier in life, such as mode of birth or breast feeding, start to converge. At around the age of 3-5 years, the microbiome resembles that of an adult, and populations stabilise.

This raises two interesting points. Firstly, if the associated health differences seen in observational studies are causally related to the microbiomic differences, the effect occurs during a very short period of time. This has been referred to as the “critical window”. Animal studies have shown that during this window, the immune system is exposed to microbial antigens via specific pattern recognition receptors (e.g., Toll-Like Receptors), and primed to tolerate these specific species that direct the immune system in later life, with evidence suggesting that the microbiota need to follow a specific sequence of colonisation to ensure an optimal cascade of immune and metabolic development^[199-204]^. Furthermore, several experiments using germ-free mice that were exposed to microbiota before weaning demonstrated less inflammation, compared to those exposed later in life^[70,205]^. Innate-like T cells appear to be crucial. These cells develop immunological effector characteristics before thymic emergence, and recent work has shown that these cells are particularly responsive to early-life antigen exposure, thereby promoting the maintenance of tissue homeostasis^[206,207]^. Early exposure to commensal microbes correlates to the abundance of mucosal-associated invariant T cells (MAIT) and NK cells, and the absence of these early life antigen exposures cannot be compensated for in later life, with work showing that these T cell populations are vital for maintaining homeostasis and preventing excessive inflammation^[206]^. As an example, one murine study showed that *Bacteroidetes* (a genus associated with breastfeeding) increased colonic CD4^+^ cytotoxic T lymphocytes and increased FoxP3 protein expression, polarising the immune system away from Th2 type allergic response, in favour of Th1-mediated anti-inflammatory IL-10 production^[208]^. Evidence in humans supports this theory of a critical window of opportunity. For instance, cohort studies have shown that children who live in rural environments have a lower prevalence of IBD and asthma in later life compared to those born in urban areas; however, this protective effect is diminished if the child moves into a rural location after the critical window (i.e., is not exposed from birth), and the protective effect is absent in adults^[148,209,210]^.

Secondly, if this critical window of opportunity during the first 3 to 5 years of life is proved to be true - and based on the available evidence, it is our opinion that this is a reasonable position to have - the highly dynamic infant microbiome offers a potential opportunity for health intervention. Building on public health advice that already exists (e.g., breastfeeding), interventions could be used to modulate the microbiome. We have discussed a Finnish group that has already shown that using soil from a forest transplanted to urban day care facilities changes the microbiome of the attendees, but large long-term studies need to be performed to see if this can influence health outcomes. Other approaches, such as using similar environmental inoculants in the home or using probiotics, have also been suggested, although the impact of this has not been assessed. Animal models are being employed in increasing numbers in an attempt to elucidate what specific microbial populations are important in orchestrating beneficial health outcomes, which in the future may be able to increase the precision of any microbiomic intervention.

## CONCLUSION

There has been a remarkable increase in knowledge over a short period of time, covering the subtleties of the infant microbiome and its variation depending on specific external stimuli. It is clear that the first five years of life are a highly complex and dynamic period for the infant microbiome. We have described how this is influenced by numerous variables in a highly orchestrated and dynamic ecosystem that follows a similar pattern across different global populations. An individual’s microbiome can be seen to diverge from birth, with CS being associated with an altered microbiome compared to VD, although the mechanism of this difference is not fully established. It is apparent that the largest single effector of the microbiome is breastfeeding, with very clear evidence that once this ceases, the microbiome of the infant quickly starts to change to that of an adult. Very few mechanistic studies have been performed to study these processes in detail. As such, observational data have been employed to infer how the environment influences the microbiome. Several studies have identified key external influences on the infant’s microbiome, but consistently breastfeeding has the largest effect size. Moreover, the available evidence suggests that as a single variable, it provides a significant and long-lasting health benefit to the infant. After breastfeeding stops, other environmental cues, such as diet, geography, and exposure to soil, start to influence the microbiome to a greater degree. This period of early life, the window of opportunity, appears key to priming the immune system and avoiding chronic inflammatory states in later life. These modifiable variables show growing promise for future clinical utility for a microbiome-based public health intervention. As our methodology improves, higher resolution analysis should make it possible to dissect specific strain-level configurations as well as the non-bacterial species of the microbiome, and the ecosystems they originate from, to enable us to fully understand how the microbiome influences the normal development of the immune and metabolic systems in humans.

## DECLARATIONS

### Copyright

© The Author(s) 2024.
